# Comparative Transcriptome Profiling Analysis Reveals the Adaptive Molecular Mechanism of Yellow-Green Leaf in *Rosa beggeriana* ‘Aurea’

**DOI:** 10.3389/fpls.2022.845662

**Published:** 2022-03-24

**Authors:** Ying Gan, Yaping Kou, Fei Yan, Xiaofei Wang, Hongqian Wang, Xiangshang Song, Min Zhang, Xin Zhao, Ruidong Jia, Hong Ge, Shuhua Yang

**Affiliations:** ^1^National Center of China for Flowers Improvement, Institute of Vegetables and Flowers, Chinese Academy of Agricultural Sciences, Beijing, China; ^2^College of Landscape Architecture and Forestry, Qingdao Agricultural University, Qingdao, China

**Keywords:** *Rosa beggeriana* ‘Aurea’, transcription profiling, leaf color mutant, mutant mechanism, differentially expressed genes (DEGs)

## Abstract

*Rosa beggeriana* ‘Aurea’ is a yellow-green leaf (*yl*) mutant and originated from *Rosa beggeriana* Schrenk by ^60^Co-γ irradiation, which is an important ornamental woody species. However, the molecular mechanism of the *yl* mutant remains unknown. Herein, comparative transcriptome profiling was performed between the *yl* type and normal green color type (WT) by RNA sequencing. A total of 3,372 significantly differentially expressed genes (DEGs) were identified, consisting of 1,585 upregulated genes and 1,787 downregulated genes. Genes that took part in metabolic of biological process (1,090), membrane of cellular component (728), catalytic (1,114), and binding of molecular function (840) were significantly different in transcription level. DEGs involved in chlorophyll biosynthesis, carotenoids biosynthesis, cutin, suberine, wax biosynthesis, photosynthesis, chloroplast development, photosynthesis-antenna proteins, photosystem I (PSI) and photosystem II (PSII) components, CO_2_ fixation, ribosomal structure, and biogenesis related genes were downregulated. Meanwhile, linoleic acid metabolism, siroheme biosynthesis, and carbon source of pigments biosynthesis through methylerythritol 4-phosphate (MEP) pathways were upregulated. Moreover, a total of 147 putative transcription factors were signification different expression, involving NAC, WRKY, bHLH, MYB and AP2/ERF, C2H2, GRAS, and bZIP family gene. Our results showed that the disturbed pigments biosynthesis result in *yl* color by altering the ratio of chlorophylls and carotenoids in *yl* mutants. The *yl* mutants may evoke other metabolic pathways to compensate for the photodamage caused by the insufficient structure and function of chloroplasts, such as enhanced MEP pathways and linoleic acid metabolism against oxidative stress. This research can provide a reference for the application of leaf color mutants in the future.

## Introduction

Leaf color mutation, such as spotted leaves, yellow-green leaves (*yls*), and striped leaves have been widely application in garden landscape. *Rosa beggeriana* Schrenk is a woody shrub of the genus *Rosa*, which is native to Xinjiang province of China, and has been used as a core cold resistant germplasm (Yang et al., 2016). *R. beggeriana* ‘Aurea’ is a *yl* mutant and originated from *R. beggeriana* Schrenk by ^60^Co-γ irradiation. It is the first *yl* mutant in the genus *Rosa* up to now and its cold resistant capability has not changed (Huang et al., 2007; Yang et al., 2016, 2020). *R. beggeriana* ‘Aurea’ was used as an important germplasm for innovation cultivars through interspecific hybridization with modern rose (Yang et al., 2016). Meanwhile, the *yl* color phenomenon can be stable to pass on to offsprings, which can be used as a screening marker for interspecies hybridization.

Leaf color variation significantly improves the horticultural value of *R. beggeriana*. However, the yellow-green mutations usually impair the photosynthetic efficiency and chloroplasts development, resulting in poor growth and photodamage in summer. The *yl* mutants are ubiquitous in higher plants, and the non-lethal mutants are ideal models for research on photosynthesis, pigment biosynthesis, chloroplast development, and nuclear–plasmid gene interaction. Plants always evolved adaptive photosynthetic strategies to survive in stress conditions. In *R*. *beggeriana* ‘Aurea,’ the *yl* mutant showed fewer number of trichomes in lower epidermis, and thinner cuticle and cytoderm thickness in upper epidermis cells, fewer number of chloroplasts, and abnormal thylakoids in palisade parenchyma cells of the leaves. Meanwhile, the photosynthesis efficiency, chlorophylls, and carotenoids accumulation were impaired (Supplementary Table S1 and Supplementary Figure S1), which resulted in photoinhibition at noon in *yl* mutant. Photorespiration was stronger and lipid peroxidation was evoking to scavenge reactive oxygen for photoprotective strategies (Yang et al., 2020). However, the underlying molecular mechanism for *yl* mutation is still unclear.

Genetic changes usually cause various leaf color types, such as albino, white emerald, light green, greenish-white, greenish-yellow, etiolation, yellow-green, and striped leaves (Zhao et al., 2020). There are more than 700 DNA mutant sites leading to leaf color change, which are mainly involved in the pigment synthesis and metabolism, chloroplast development, photosynthesis, and their signal transduction, etc. Leaf color mutants have been reported in many species: *Zea mays*, *Populus* L., *Nicotiana tabacum*, *Arabidopsis thaliana*, *Oryza sativa*, *Cucumis melo*, *Forsythia koreana*, *Cymbidium* (Kim et al., 2020), *Lagerstroemia indica*, and *Ginkgo biloba* L. (Liu et al., 2016). However, the underlying molecular mechanisms of leaf color formation are different and poorly understood.

In recent years, a high-throughput RNA-sequencing (RNA-Seq) technology has been widely applied for understanding the regulation mechanism of specific biological functions (Zhang et al., 2014; Bellieny-Rabelo et al., 2016). Several leaf color mechanisms have been demonstrated *via* RNA-Sequence in some ornamental plants. In *Paeonia suffruticosa*, the comparative transcriptome analysis of three types of leaf color in Tree peony showed that chlorophyll and carotenoid biosynthesis, flavonoid/anthocyanin biosynthesis relative genes with significantly different expression during leaf color changes. Eight genes were predicted to participate in anthocyanin biosynthesis, eight genes were predicted to be involved in porphyrin and chlorophyll metabolism, and 10 genes were predicted to participate in carotenoid metabolism. Those genes may be mainly responsible for purplish red leaf color and yellowish green leaf color in spring (Luo et al., 2017). In *Lagerstroemia indica*, analysis of the differentially expressed genes (DEGs) between mutated-yellow green and original-green leaves illustrated that the leaf color formation was greatly affected by the transcription level of genes involved in chloroplast development and chlorophyll metabolism, phototransduction pathway, the plant–pathogen interaction pathway, and photosystem. In addition, RNA sequencing is used to study leaf mutation mechanism in *Cymbidium*, such as yellow leaf color mutation of *Cymbidium longibracteatum* (Jiang et al., 2020), striped leaf mutation of *Cymbidium sinense* ‘Dharma’ (Zhu et al., 2015), and yellow leaf color mutation of *Cymbidium* sp. (Kim et al., 2020). All the results of the transcriptome research provide us new insights into the molecular mechanism of leaf color mutation.

In this study, the comparative transcriptome profiling was carried out between the *yl* type and normal green color leaf type (WT) by RNA sequencing. The density of trichome and stomatal in the epidermis and the ultrastructure of chloroplast were confirmed through a scanning electron microscope (SEM). The chlorophyll fluorescence and pigment contents were determined to provide proofs for our RNA-seq results. We aimed to reveal the mutation mechanism of *R. beggeriana* ‘Aurea’ and provide a deep insight into its adaptive strategies. The transcriptome database provides a valuable reference for the genetic engineering of leaf color variant in *R. beggeriana* and other plant species.

## Materials and Methods

### Plant Material

**FIGURE 1:**
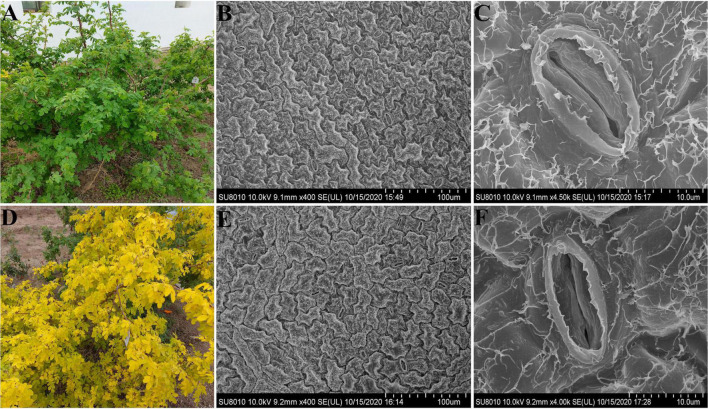
Phenotype and leaf epidermis ultrastructures of wild type (WT) and yellow-green leaf (*yl*) mutant. Panels **(A–C)** present WTs. Panels **(D–F)** present *yl* mutants. Panel **(A)** is *Rosa beggeriana*, used as WT. Panel **(D)** is the *yl* mutant. Panels **(B,E)** are leaf lower epidermis in bar with 100 μm. Panels **(C,D)** are leaf stomata in bar with 10 μm.

The *R. beggeriana* ‘Aurea’ mutant (*yl*) was obtained from wild type (WT) *R. beggeriana* (green leaf) (Figures 1A,D) by ^60^Co-γ irradiation treatment. Compared with WT, the *yl* mutant leaf displayed a yellow-green color during the entire growth cycle. The yellow-green color was relatively easy to distinguish from WT, but yellow-green color was easily affected by light intensity. The yellow-green color leaves from the same mutant plant would exhibit more green color under shading conditions (Supplementary Figures S4B,C).

In this study, we used the leaves of *R. beggeriana* ‘Aurea’ and *R. beggeriana* grafting plantlets as experimental materials, ‘Dancing Butterfly’ was used as rootstock. All of them were grown in the National Center of China for Flowers Improvement, Institute of Vegetables and Flowers of the Chinese Academy of Agricultural Sciences, Beijing, China. Leaves were collected from the normal yellow-green color leaves for RNA-Seq and SEM observation. Leaves for RNA-Seq were frozen in liquid nitrogen and stored at −80°C until used.

### RNA Extraction, Library Construction, and RNA Sequencing

A total of six independent samples were measured, such as *R. beggeriana* leaves (WT-1, WT-2, and WT-3) and *R. beggeriana* ‘Aurea’ leaves (*yl*-1, *yl*-2, and *yl*-3). Total RNA was extracted using the RNAprep pure Plant Kit (Tiangen, Beijing, China) according to the manufacturer protocol and treated with RNase-free DNase I to prevent genomic DNA contamination, and the quantity and quality of the extracted RNAs were assessed by using the NanoDrop ND2000 spectrophotometer (Thermo Scientific, MA, United States) and 1% (w/v) agarose gel electrophoresis. cDNA libraries constructions and transcriptome sequencing were performed by the Biomarker Technologies Co., Ltd. (Beijing, China). mRNAs of each sample were enriched and purified with oligo (dT)-rich magnetic beads and then randomly fragmented into small pieces. Using these short fragments as templates to synthesize the first strand cDNA by random hexamer primers and the second strand cDNA subsequently synthesis by RNase H and DNA polymerase I. The double-strand cDNA was subjected to an end repaired, poly (A) added and sequencing adapters connected. The appropriate length of cDNA fragments was separated by agarose gel electrophoresis and amplified by PCRs to make the cDNA libraries. The quality of the sample libraries was determined by an Agilent 2100 Bioanalyzer and Qubit2.0. Finally, the well-constructed library was sequenced using an Illumina HiSeq™ 2500 platform (Beijing Biomarker Technology, Beijing, China).

### *De novo* Assembly and Gene Annotation

Clean reads were obtained by filtering out adaptor-only nucleotides using Trimmomatic (v 0.32). Then, clean reads were mapped to the reference genome *Rosa chinensis* ‘Old Blush’^1^ by Tophat2 (Kim et al., 2013). The Q30, GC-content, and sequence duplication level were calculated. HMMER software was used to obtain the annotation information of the unigenes by comparing with the Pfam database (Bateman et al., 2004). A gene name was assigned to each protein sequence based on the best BLAST hit. All the unigenes were annotated *via* comparisons to the non-redundant protein sequence (Nr) database (Deng et al., 2006), Swiss-Prot database (Apweiler et al., 2004), Gene Ontology (GO) database (Ashburner et al., 2000), and Kyoto Encyclopedia of Genes and Genomes (KEGG) databases (Kanehisa et al., 2004) using BLASTp with *E*-values < 10^–5^.

### Differentially Expressed Genes Analysis

To identify DEGs between *yl* and WT, the Cufflinks was used to calculate gene abundance based on fragments per kilobase of transcript per million mapped reads (FPKM) (Trapnell et al., 2010). Then, the DESeq R (1.10.1) package was used to identify the DEGs with a model based on the negative binomial distribution (Wang et al., 2010). The false discovery rate (FDR) < 0.01, and log-fold expression change (log FC) of ≥2 found by DESeq were assigned as differentially expressed. The FDR of DEGs was adjusted by the Benjamini and Hochberg’s approach (Anders and Huber, 2010). These DEGs carried out GO functional enrichment analysis and KEGG pathway analysis by the GOseq R Packages and KOBAS software, respectively (Mao et al., 2005; Young et al., 2010).

### Real-Time Quantitative PCR Verification and Expression Analysis

A total of 30 DEGs were selected and analyzed by the real-time quantitative PCR (qRT-PCR) (Bio-Rad, CA, United States) using the SYBR Premix Ex Taq™ II kit (Takara Biomedical Technology, Beijing, China). The gene-specific primers were designed using the software Primer Premier 5.0 and listed in Supplementary Table S2. The *Rb*α*-Tubulin* was used as internal control genes and the relative expression was calculated by the 2^–△△*Ct*^ method (Livak and Schmittgen, 2001). The statistical analysis was performed by IBM SPSS Statistics 2019. The statistical significance was defined as *p* < 0.05. Three independent biological replications and three technical replications were used, respectively.

### Electron Microscopy and Morphometric Evaluation

To evaluate the effects of leaf mutation on chloroplast structure, the 2 mm × 2 mm pieces from uppermost leaf on the sunny side of an annual branch from WT and *yl* mutant plants were cut from leaves for SEM (Figures 1B,C,E,F). Then the leaf cut sections were fixed in 2.5% glutaraldehyde and 1% osmium tetroxide, dehydrated in a series of graded ethanol as previous description (Yang et al., 2020). The samples were observed and photographed with a SEM SU-8010 (Hitachi Ltd., Tokyo, Japan). Then, the stomatal density, chloroplast ultrastructure were measured and analyzed according to the previous description (Yang et al., 2020).

### Chlorophyll Fluorescence Measurements

Chlorophyll fluorescence parameters were measured as previous description (Yang et al., 2020) with a portable fluorometer DAUL-PAM-100 (Walz, Effeltrich, Germany) at 12:00. The relative parameters of *F*_*V*_/*F*_*M*_, Y(II) photosystem II (PSII), ETR, Y(NO), Y(NPQ), qN, qP, and qL were calculated and analyzed (Liu et al., 2019).

## Results

### The Mutant Hindered the Size and Numbers of Stomata

The yellow-green color leaf is easily identifiable and has ornamental value in city landscape (Figures 1A,D). In this research, *yl* mutant presented lower pigment contents (Supplementary Figure S1) and chlorophyll fluorescence content (Supplementary Table S1), thinner leaf cross-section, fewer chloroplasts, and abnormal chloroplast structure (Supplementary Figures S2, S3), which were consistent with our former research. Stomata play an important role on photosynthesis, previous researchers indicated that Chlorophyll-deficient mutants improve thermal dissipation *via* stomatal regulation to the avoidance of photoinhibition (Gang et al., 2019). In this study, the conditions of stomata were measured through an SEM (SEM) between *yl* mutant and WT. Our results showed that the stomatal had normal apparatus and consist of two guard cells and two accessory guard cells. Both of them have epidermal hairs, but the WT has larger stoma and higher stoma density compared with the *yl* mutant (Figures 1B,C,E,F and Supplementary Figure S3). These implied that the heat dissipation *via* stoma was impaired in *yl* mutant.

### Functional Annotation and Classification of Unigenes

A total of 155,542,064 clean reads were generated, with 77,273,505 reads from the WT and 78,268,559 from the *yl* mutation, where the GC content ranged from 47.29 to 48.08%, and the Q30 percentage exceeded to 90.01%. The RNA-seq read data have been submitted to the NCBI Sequence Read Archive (NCBI SRA) under accession number PRJNA798584. The high-quality clean reads of each sample were mapped to the reference genome *Rosa chinensis* ‘Old Blush’ (Raymond et al., 2018), and the mapping efficiency ranged from 56.83 to 62.00% (Supplementary Table S3).

**FIGURE 2:**
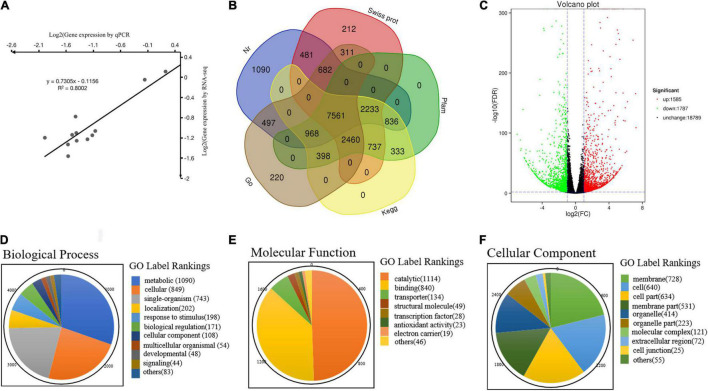
Annotation information and functional annotation of DEGs in WT and *yl* mutant. **(A)** Correlation analysis of the RNA-seq data. **(B)** Venn diagram of the distribution of annotation information from different public databases. **(C)** The volcano plot of DEGs. **(D)** Biological process. **(E)** Molecular function. **(F)** Cellular component.

The BLAST alignment was utilized to annotate the unigenes in our transcriptome. A total of 20,619 unigenes were identified and annotation with an *E*-value threshold of 1 × 10^–5^ in the major public databases, such as Nr (14,347), KEGG (8,232), Pfam (15,525), GO (13,096), and Swiss Prot (14,676) (Supplementary Tables S4–S7). Above all, 7,561 (36.7%) unigenes shared annotation in all databases, and 19,499 (94.5%) unigenes were successfully annotated in at least one database. There were 15,526 unigenes with significant matches in the Pfam database, accounting for the highest proportion (75.2%), while the lowest proportion (8,233; 39.9%) was obtained from the KEGG database (Figure 2B).

### Real-Time Quantitative PCR Analysis of Differentially Expressed Genes

**FIGURE 3:**
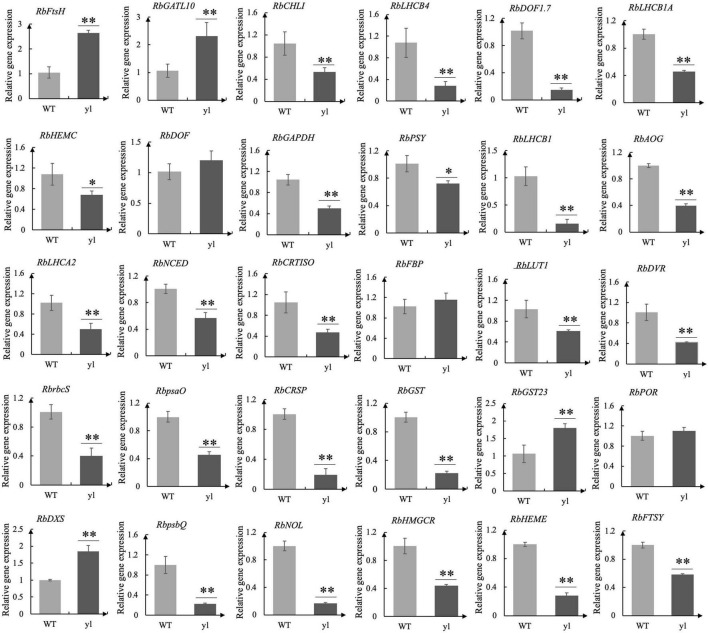
Real-time quantitative PCR (qRT-PCR) analysis for 30 differentially expressed genes (DEGs). The qRT-PCR were performed for three biological replicates and three technical repetitions. ^**^indicates very significant difference of gene expression at 0.01 level. *indicates significant difference of gene expression at 0.05 level.

To verify the reliability of the transcriptome data, qRT-PCR was used to prove the DEGs between WT and *yl*. Through transcriptome analysis, some key DEGs associated with *yl* formation have been identified and qRT-PCR analysis was performed (Supplementary Table S8). These genes involved in chloroplast development, stomatal structure, chlorophylls metabolism, Photosystem I (PSI), PSII, carotenoids metabolism, MVA pathway, MEP pathway, stomatal development, and antioxidants. A total of 30 DEGs were selected for qRT-PCR analysis and the test results are consistent with the transcriptome data (Figure 3 and Supplementary Tables S2, S8). The relative expression level estimated by RNA-seq and qRT-PCR were strongly correlated (*R*^2^ = 0.815) (Figure 2A). The results indicated that the transcriptome data were reliable and could be used for further research.

### Analysis of Differentially Expressed Genes

A total of 3,372 DEGs with 1,585 upregulated and 1,787 downregulated, respectively (Figure 2C and Supplementary Table S5). According to GO functional analysis, a total of 1,867 DEGs were selected from all 23,560 unigenes, classified into three major GO term categories: biological process, cellular component, and molecular function. The three major GO term categories were divided into 43 subcategories. In the 43 subcategories of the GO classification, metabolic process (GO:0008152), cellular process (GO:0009987), and single organism (GO:0044699) were the most highly represented GO terms in the biological process category (Figure 2D); catalytic activity (GO:0003824) and binding (GO:0005488) showed the highest numbers of DEGs for the molecular function category (Figure 2E); membrane (GO:0016020), cell (GO:0005623), and cell parts (GO:0044464) were the majority of group in the cellular component category (Figure 2F). Meanwhile, only a few DEGs were assigned to biological adhesion (GO:0022610), immune system process (GO:0002376), nucleoid (GO:0009295), and translation regulator activity (GO:0001071). No DEGs were assigned to cell killing (GO:0001906), locomotion (GO:0040011), rhythmic process (GO:0048511), extracellular region part (GO:0005576), metallochaperone activity (GO:0016530), and protein tag (GO:0031386) subcategories (Supplementary Table S6). These results indicated that the cell membrane of cellular component, the metabolic pathway of biological process, and the catalysis activity of molecular function were altered in *yl* mutant. The change of metabolism pathway that occurs in cell membrane maybe the main reason for yellow-green phenotype in *yl* mutants.

**FIGURE 4:**
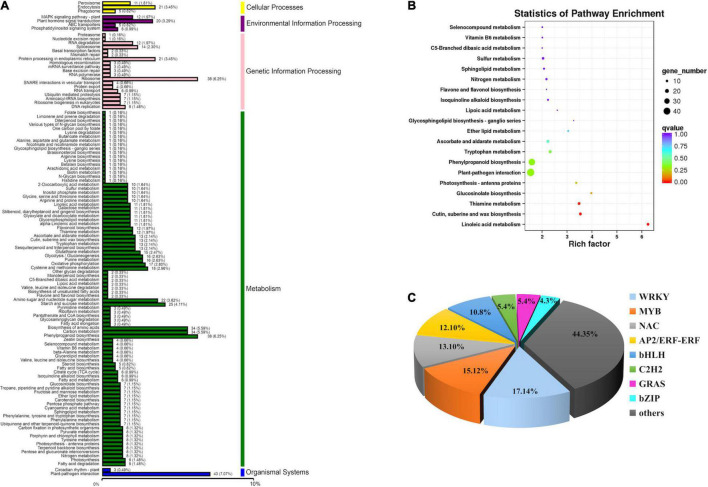
Function annotation of DEGs. **(A)** Classification statistics of Kyoto Encyclopedia of Genes and Genomes (KEGG) pathway annotation. **(B)** The KEGG enrichment analysis of DEGs. **(C)** DEGs involved in transcription factors.

Kyoto Encyclopedia of Genes and Genomes mapping showed that a total of 7,943 unigenes were classified into 118 metabolism pathways, 608 genes were selected to present the system biological function of DEGs between *yl* and WT. KEGG classification showed that most of the DEGs were enriched in metabolism and genetic information processing. Among them, phenylpropanoid biosynthesis (38 DEGs, 6.25%), carbon metabolism (34 DEGs, 5.59%), biosynthesis of amino acids (34 DEGs, 5.59%), starch and sucrose metabolism (25 DEGs, 4.11%), and amino sugar and nucleotide sugar metabolism (22 DEGs, 3.62%) accounted for the main metabolism pathways; ribosome (38 DEGs, 6.25%) pathway represented the largest proportion in genetic information processing. Besides, the plant–pathogen interaction pathway (43 DEGs, 7.07%) in organismal systems and plant hormone signal transduction pathway (20 DEGs, 3.29%) in environmental information processing also take large percentage in KEGG pathway (Figure 4A). Those results implied that metabolism and genetic information processing related genes were mainly responsible for leaf color alternative in *yl* mutants. Especially, the ribosome pathway related genes may be involved in nuclear–plasmid interaction, as many ribosomal proteins had significantly different expression that were located in chloroplastic (Supplementary Table S7).

To further understand the KEGG pathway, we carried out the enrichment analysis of KEGG Pathway. The results showed that phenylpropanoid biosynthesis (ko00940) and plant–pathogen interaction (ko04626) enriched the most member of DEGs, 38 DEGs and 43 DEGs were clustered, respectively. As shown in Table 1, when *p* was used for represented the enrichment of KEGG pathway, the top ten KEGG pathway enrichments were linoleic acid metabolism (ko00591), cutin, suberine and wax biosynthesis (ko00073), thiamine metabolism (ko00730), glucosinolate biosynthesis (ko00966), photosynthesis-antenna proteins (ko00916), plant–pathogen interaction (ko04626), phenylpropanoid biosynthesis (ko00940), tryptophan metabolism (ko00380), ascorbate and aldarate metabolism (ko00053), and ether lipid metabolism (ko00565). When rich factor was used to describe the enrichment efficiency, linoleic acid metabolism (ko00591, 6.25), glucosinolate biosynthesis (ko00966, 3.98), cutin, suberine, and wax biosynthesis (ko00073, 3.54), thiamine metabolism (ko00730, 3.48), photosynthesis-antenna proteins (ko00916, 3.37), glycosphingolipid biosynthesis-ganglio series (ko00604, 3.27), and ether lipid metabolism (ko00565, 3.05) were the most clustered pathways (Figure 4B and Supplementary Table S7). In our former research, the photosynthetic efficiency was impaired and the cuticle, cytoderm thickness were thinner in *yl* plants. Here, photosynthesis-antenna proteins and cutin, suberine, and wax biosynthesis pathway related genes may be responsible for the lower photosynthesis and thinner cuticle phenotype.

**TABLE 1:** Top 10 of Kyoto Encyclopedia of Genes and Genomes (KEGG) enrichment pathway.

KEGG pathway	Ko id	*P*-value	*Q*-value	Cluster frequency
Linoleic acid metabolism	ko00591	2.79E-07	3.29E-05	11/608, 1.81%
Cutin, suberine, and wax biosynthesis	ko00073	4.29E-05	0.00507	13/608, 2.14%
Thiamine metabolism	ko00730	9.96E-05	0.0117	12/608, 1.97%
Glucosinolate biosynthesis	ko00966	0.00123	0.145	7/608, 1.15%
Photosynthesis - antenna proteins	ko00196	0.00183	0.216	8/608, 1.32%
Plant–pathogen interaction	ko04626	0.00262	0.3092	43/608, 7.07%
Phenylpropanoid biosynthesis	ko00940	0.00269	0.317	38/608, 6.25%
Tryptophan metabolism	ko00380	0.00330	0.385	13/608, 2.14%
Ascorbate and aldarate metabolism	ko00053	0.00467	0.551	13/608, 2.14%
Ether lipid metabolism	ko00565	0.00640	0.755	7/608, 1.15%

### Antioxidant System Was Activated

When plants suffer high light stress, ascorbate-glutathione cycle can be evoked to alleviate oxidative stress by regulating the cellular redox status (Yang et al., 2020). Thiamine, also known as vitamin B1, is known to play a fundamental role in energy metabolism. Phenylpropanoids and glucosinolates are the two classes of secondary metabolites, they can help plants survive during stress conditions (Kim et al., 2019). Glucosinolates that are derived from tyrosine and phenylalanine are called aromatic glucosinolates, whereas glucosinolates derived from tryptophan or from aliphatic amino acids are called indole or aliphatic glucosinolates, respectively (Giuliano et al., 2008). In this study, linoleic acid metabolism, ascorbate and aldarate metabolism, ether lipid metabolism, and tryptophan metabolism were significantly clustered (Figure 4B), which implied that antioxidant system may be activated to compensate for the adverse situation *via* lipid peroxidation. This result was consistent with our former research.

### Photosynthesis and Chloroplast Development Related Genes Were Downregulated

**FIGURE 5:**
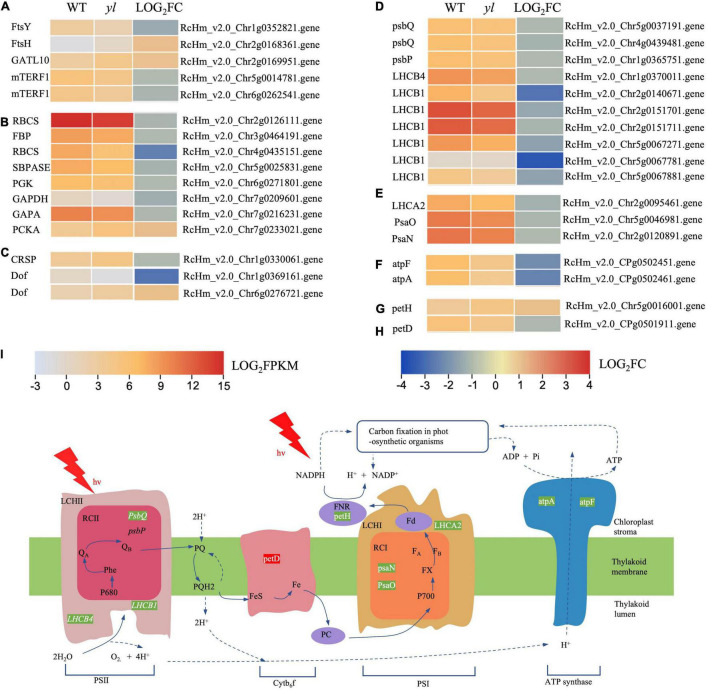
The expression profiles of DEGs involved in chloroplast development and function. **(A)** DEGs involved in chloroplast division and development. **(B)** Expression profiles of DEGs involved in carbon fixation in photosynthetic organism photosynthesis metabolism. **(C)** DEGs involved in stomatal formation and development. **(D)** DEGs involved in inorganic ion transport and metabolism. **(E)** DEGs involved in Cytb_6_f metabolism. **(F)** DEGs involved in photosystem II (PSII). **(G)** DEGs involved in photosystem I (PSI). **(H)** DEGs involved in F-type ATPase metabolism. **(I)** Simplified scheme for the thylakoid electron transport chain. Green letters indicate that the transcription level of genes in *yl* was lower than WT. Red letters indicate that was higher than WT. mTERF1, mitochondrial transcription termination factors; RBCS, ribulose-bisphosphate carboxylase small chain; SBPASE, sedoheptulose-bisphosphatase; PGK, phosphoglycerate kinase; GAPA, glyceraldehyde-3-phosphate dehydrogenase (NADP+); PCKA, phosphoenolpyruvate carboxy kinase; petH, ferredoxin–NADP+ reductase; petD, cytochrome b6-f complex subunit 4; psbP, photosystem II oxygen-evolving enhancer protein 2; PsaN, photosystem I subunit PsaN; atpF, F-type H+ -transporting ATPase subunit b; atpA, F-type H+ -transporting ATPase subunit b.

Yang et al. (2020) showed that the chloroplast ultrastructure and photosynthesis were impaired and then resulted in photoinhibition at noon in the *yl* mutant. The *yl* mutant preferred to consume the excited energy *via* photorespiration and lipid peroxidation to scavenge reactive oxygen for subsistence during high light conditions (Yang et al., 2020). To further investigate the molecular mechanism of this adaptive strategies, the photosynthesis and chloroplast development related genes were comparatively analyzed. A total of 16 genes have been significantly different expression between *yl* and WT, and their expression heatmap are indicated in Figure 5 and Supplementary Table S8.

Chloroplast is composed of chloroplast membrane, thylakoid, and stroma, it is the place for the synthesis of crucial photosynthetic pigments and photosynthesis. The thylakoid membrane is inlaid with proteins, pigments, and other components of the five functional complexes which directly related to photosynthesis (Powell et al., 2012). The complexes contain PSII; cytochrome f/b6 (cytf/b6); PSI; chlorophyll a/chlorophyll b light-harvesting complex (LHC) and ATP synthase (Llorente et al., 2020). PSII is mainly distributed in the stacked part of the thylakoid membrane. The function of PSII is to absorb the solar energy to oxidize water and reduce plastoquinone (Lima et al., 2018). PSI is responsible for transferring electrons to ferredoxin (Fd), under the action of reductase, nicotinamide adenine dinucleotide phosphate (NADPH) is formed by NADP^+^ and Fd (Ali et al., 2019). Mutations of genes related to the chloroplast development and photosystem can cause yellow leaf color, reduce the efficiency of photosynthesis, slow plant growth, and cause plant death (Latijnhouwers et al., 2010; Armbruster et al., 2011). *PsbQ*, *PsbP*, *LHCB1*, and *LHCB4* were responsible for encoding the PSII reaction center proteins, and PsaN, PsaQ, and LHCA2 were important proteins for PS I, *atpA*, and *atpF*, encoded F-type ATPase, all of them have decreased expression in *yl* than WT (Figures 5D–I). Other transcripts HCF136 and HCF101, which are required for the translation of three PSII subunit genes *PsbH*, *PsbT*, and *PsbB*. The cytb6f complex genes, *petM* and *petJ*, have no differential expression. However, one of the cytb6f complex genes, *petH* has increased transcription in *yl* mutant (Figure 5I). *RBCS*, *FBP*, *SBP*, *PGK*, *GAPDH*, and *PCKA* are important genes for carbon fixation in photosynthetic organisms. Excluding *PCKA*, the transcription levels of them were significantly depressed in *yl* mutant (Figures 3, 5B,I). The down-expression of those genes implied that photosynthesis efficiency may be impaired in *yl* mutant.

The *FtsH* and *FtsY* genes are essential for the morphogenesis and stability of chloroplasts, the decrease of *FtsH* gene expression will inhibit the formation of chloroplast thylakoids (Kogata et al., 1999; Takechi et al., 2000; Sakamoto et al., 2003). Glutamine amidotransferase (GATL) catalyzes the removal of the ammonia group from glutamine and then transfers this group to a substrate to form a new carbon-nitrogen group. *AtGATL12* plays an important role in the chloroplast development in *Arabidopsis*, the deletion of *AtGATL12* gene causes yellow flowers in seedlings (Su et al., 2011). Mitochondrial transcription termination factors (*mTERF* protein family) are important regulators of mitochondrial gene transcription, which can regulate the expression of organelle genes at different levels (Yin et al., 2021). In *Arabidopsis*, the mutant of *mTERF9* gene with T-DNA insertion showed obvious yellowing color leaf phenotype, which demonstrate that *mTERF9* may be involved in the regulation of chloroplast development in *Arabidopsis* (Meteignier et al., 2021). In this study, the expressions of *FtsY* and *mTERF1* were downregulated, while the expressions of *FtsH* and *GATL10* were upregulated (Figures 3, 5A). These results implied that the development of chloroplast was disturbed and may resulted in *yl* color. This was agreed with former research that a smaller number of chloroplasts in *yl* mutant.

The development of chloroplasts requires the coordination of chloroplast genes and nuclear genes. PPR protein is the key component of the editosome, which was responsible for chloroplast RNA editing site recognition and binding. Chloroplast ribosomal protein genes (RP), such as *RPL* and *PRS* play intrinsic roles in the structural stability of the ribosomal complex (Ishii et al., 2006). In addition, they are crucial for the assembly and development of chloroplast (Degenhardt and Bonham-Smith, 2008). In our study, a number of chloroplastic location PPR protein genes were obviously altering in *yl* mutant, implied that chloroplast development defect may be caused by PPR proteins and RPL protein genes (Figure 3 and Supplementary Table S8).

Above all, our results suggested that the abnormal chloroplast structure impaired photosynthesis. As the chlorophylls synthesis is localized at chloroplast stroma, chloroplast membrane, and thylakoid membrane, the destruction of chloroplast structure may affect the accumulation of chlorophylls and carotenoids. The disorder proportion of chlorophylls and carotenoids can also result in *yl* color.

### Chlorophylls Biosynthesis Related Genes Were Downregulated

**FIGURE 6:**
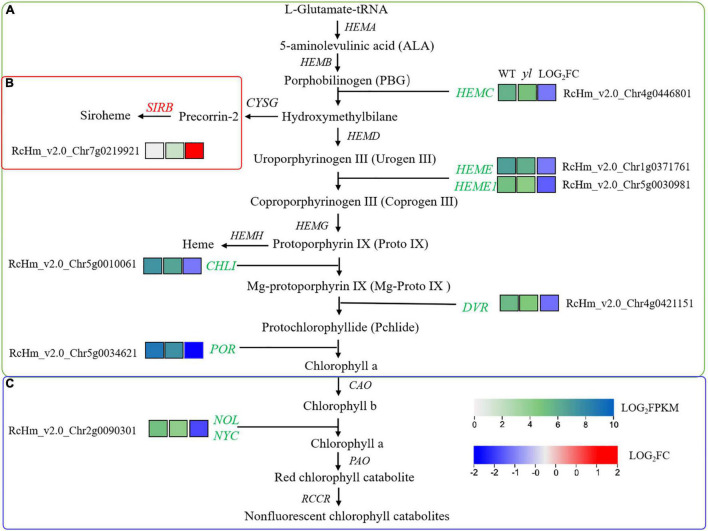
Differentially expressed genes (DEGs) involved in chlorophyll biosynthesis. **(A)** Chlorophyll biosynthesis pathway. **(B)** Siroheme biosynthesis pathway. **(C)** Chlorophyll degradation pathway. SIRB, siro hydro chlorin ferrochelatase.

As a vital components of photosystem light-harvesting antenna complexes, chlorophyll a and chlorophyll b make more efficient transport of the energy of absorbed light toward the light reaction center (Tanaka and Tanaka, 2006). In early research, the accumulation of chlorophylls and carotenoids are decreased in *yl* mutant (Yang et al., 2020). To better understand the molecular mechanism of *yl* color, the biosynthesis and degradation of chlorophylls, siroheme and heme biosynthesis were investigated in our study (Figures 3, 6 and Supplementary Table S8). Chlorophylls biosynthesis is a complex process and involves in many enzymes in green plants (Beale, 2005). From the beginning of glutamyl-tRNA (glu-tRNA) to the end of Chlorophyll b synthesis, there are 16 steps, which are completed by 16 enzymes encoded by more than 20 genes (Beale, 2005). Any mutation of the related gene in this pathway could affect the synthesis of chlorophylls, and results in leaf color variation. In *Arabidopsis*, *HEMC* catalyzes porphobilinogen to produce hydroxymethyl. The knockdown of *HEMC* showed an albino and seedling-lethal phenotype, and the expression levels of the *psaA*, *psaB*, and *atpB* genes (PEP- and NEP-dependent genes) were reduced by approximately 15–20% compared with WT plants (Huang et al., 2017). *HEME* encoding uroporphyrinogen decarboxylase, the downregulation of *HEME* will trigger the inhibition of chlorophyll biosynthesis and chloroplast development, which may eventually lead to gradual chlorosis and cell death in wheat (Jiang et al., 2013). Mg-chelatase is the first enzyme in the magnesium branch of the chlorophyll biosynthetic pathway. It is composed of three subunits, namely, the I subunit (CHLI), the D subunit (CHLD), and the H subunit (CHLH). In the *CHLI* silence pea plants, the leaves, stem, calyces and pod turn yellow, and the contents of photosynthesis relative pigments decrease strongly (Adhikari et al., 2009). 8-Vinyl reductase (DVR) is indispensable for non-vinyl chlorophyll synthesis, in rice, the deletion mutant of *DVR* exhibited a *yl* phenotype, arrested chloroplast development, reduced chlorophyll– level, and impeded growth (Adhikari et al., 2009). NADPH protochlorophyllide oxidoreductase (POR), which catalyzes the conversion of protochlorophyllide into chlorophyllide, is the only light-requiring reaction in the chlorophyll synthesis pathway (Griffiths, 1978). In *Arabidopsis*, the *porb* and porc double mutants have the phenotype of lethal chlorosis at the seedling stage (Tatsuru et al., 2003). The decreased expression of *OsPORA* and *OsPORB* causes light green leaf color in the early stage of seedling, and then yellow spots or white spots in rice leaf (Sakuraba et al., 2013). In higher plant, *NOL/NYC* encoding chlorophyll b reductase enzyme that can catalyze the first step of the conversion of chlorophyll b to chlorophyll a, the overexpression of *NOL/NYC* might promote the degradation of chlorophyll b to chlorophyll a and affect chlorophyll biosynthesis (Li et al., 2018).

Our results showed that the transcription levels of *HEMC*, *HEME*, *CHLI*, *DVR*, and *POR* genes that involved in chlorophyll synthesis were all downregulated expression in *yl* mutant (Figures 3, 6A). *NYC1* and *NOL* involved in chlorophylls degradation were also significantly down-expressed (Figures 3, 6C). These results implied that both the biosynthesis and degradation of chlorophylls were depressed, and then reduced the accumulation of chlorophylls in *yl* mutant. On the contrast, the expression of *SIRB* genes that involved in siroheme synthesis was upregulated in *yls* compared with those in green leaves (Figures 3, 6B). As a branched pathway of modified tetrapyrroles, the biosynthesis of siroheme is a competitor in synthetic precursors for chlorophyll a and b (Bali et al., 2011). The increase expression levels of *SIRB* may cause the reduction of the chlorophyll a/b biosynthesis and accumulation in yl mutants.

### Methylerythritol 4-Phosphate Pathway Was Enhanced and Mevalonate Pathway Was Depressed

**FIGURE 7:**
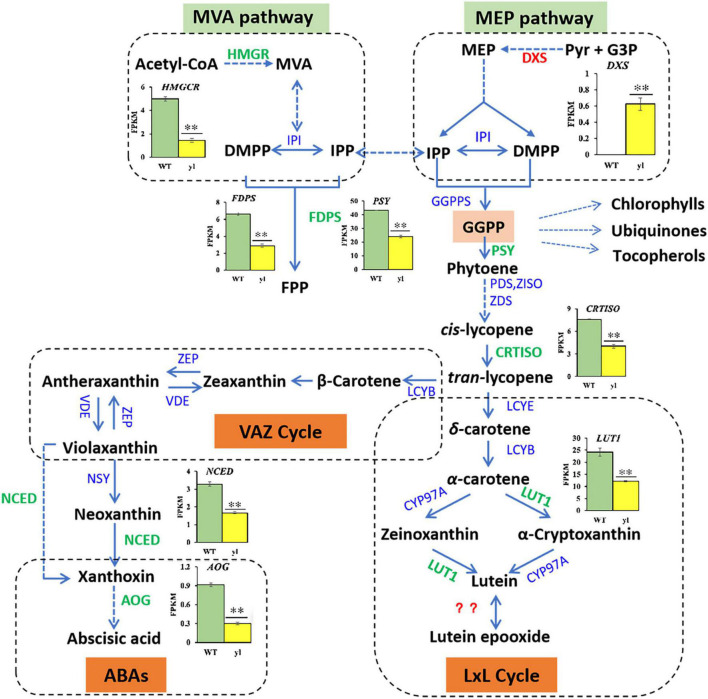
Differentially expressed genes (DEGs) related to carotenoid biosynthesis. FDPS, 1-deoxy-D-xylulose-5-phosphate synthase; NCED, 9-*cis*-epoxycarotenoid dioxygenase; AOG, abscisate beta-glucosyltransferase.

Carotenoids are natural terpenoid pigments and play a critical role on the plants adaption of different light conditions and changing environments. Acting as antenna pigments in the reaction center of PS II, carotenoids transfer the captured light energy to chlorophylls. We usually regulated the transcription levels of the enzymes and genes involved in carotenoids biosynthesis to regulate the type and quantity of carotenoids productions. Isoprene pyrophosphate (IPP) is the precursor of carotenoids, which is produced *via* mevalonate (MVA) pathway and the methylerythritol 4-phosphate (MEP) pathway, independently. In the MVA pathway, 3-hydroxy-3-methylglutaryl coenzyme A reductase (HMGR) is the rate-limiting step and catalyzes the conversion of HMG-CoA to MVA (Narita and Gruissem, 1989). 1-Deoxy-D-xylulose 5-phosphate synthase (DXS) is the first rate-limiting enzyme in the MEP pathway to form IPPs. An overexpression of *DXS* gene can increase the levels of different terpenoids in *Arabidopsis* (Estevez et al., 2001). In this study, the transcription level of DXS was remarkably increased in *yl* mutant, where *HMGR* was decreased (Figures 3, 7 and Supplementary Table S8). These results implied that MEP pathway was enhanced and MVA pathway was inhibited in *yl* mutant. For the content of total carotenoids was reduced, so the detail steps of carotenoids biosynthesis pathway were investigated as showed in Figure 7.

### Xanthophyll Cycle Was Impaired During Carotenoid Biosynthesis

Carotenoids are produced from IPP and dimethylallyl diphosphate (DMAPP) *via* MEP pathway in plastids. Phytoene synthase (PSY) catalyzes the condensation of two geranylgeranyl pyrophosphate (GGPP) molecules to generate phytoene, which is the foremost and the major rate-limiting step in carotenoids biosynthesis (Cazzonelli and Pogson, 2010). Several metabolite groups, such as chlorophylls, ubiquinones, and tocopherols are also synthesized from GGPP, which will former the branch point in pathways (Vranová et al., 2012). Following, all-*trans*-lycopene produced by an array of sequential desaturations reactions by phytoene denature (PDS), ζ-carotene isomerase (Z-ISO), ζ-carotene desaturase (ZDS), and carotenoid isomerase (CrtISO) were catalyzed. Then, a critical branch-point in carotenoid biosynthesis by lycopene ε-cyclase (LYCE) and lycopene β-cyclase (LYCB) catalytic cyclization, which leads to the production of α-carotene and β-carotene, respectively. Both α-carotene and β-carotene undergo consecutive hydroxylation and then result in the production of zeaxanthin and lutein, respectively. During the hydroxylation of zeaxanthin, β-cryptoxanthin is an intermediate product. Zeaxanthin undergoes epoxidation and de-epoxidation with the catalyzation of zeaxanthin epoxidase (ZEP) and violaxanthin deep oxidase (VDE) to synthesize antheraxanthin and violaxanthin. The conversation between zeaxanthin and violaxanthin former the violaxanthin-antheraxanthin-zeaxanthin cycle (VAZ cycle), and its mechanism is helpful to protect plants from photooxidative damage (Jahns and Holzwarth, 2012). During the hydroxylation of lutein, LUT1 acts as a carotene ε-hydroxylase to transform zeaxanthin into lutein. In plants with the lutein epoxide (LxL cycle), lutein can be converted into lutein epoxide (and vice versa) by particular enzymes. However, the identity of the enzyme and the mechanism of action remain unclear (Concepcion et al., 2018). The conversion of violaxanthin into 9-*cis*-neoxanthin by the enzyme neoxanthin synthase (NXS) is the final step of carotenoids biosynthesis (Neuman et al., 2014). In addition, neoxanthin can be used as precursors of phytohormones, such as ABA, which are ordinally catalyzed by 9-*cis*-epoxycarotenoid dioxygenase (NCED) and abscisate beta-glucosyltransferase (AOG).

The *PSY* and *CRIISO* play important roles in carotenoids biosynthesis. PSY is considered to be the most key regulatory enzyme in the carotenoid synthesis pathway (Cazzonelli and Pogson, 2010). In *Arabidopsis*, an overexpression of *PSY* can lead to a substantial increase in the content of β-carotene in the leaves, and a corresponding increase in the total amount of carotenoids (Toledo-Ortiz et al., 2010). The *CRTISO* gene catalyzes the formation of *cis*-lycopene from *trans*-lycopene and the depressed expression of *CRTISO* causes the accumulation of *cis*-lycopene, which ultimately leads to *yls* in Nicotiana tabacum (Shi et al., 2014). Blockage of the pathway can result in an albino phenotype and a developmental block in the light. In *yl* mutant, *PSY* (RcHm_v2.0_Chr3g0488531) and *CRTISO* (RcHm_v2.0_Chr6g0289711) were all down-expression, which may lead to *yl* color in *yl* mutant (Figures 3, 7 and Supplementary Table S8).

Moreover, carotenoids are essential for photoprotection against photooxidative damage through dissipating the excess energy in the form of heat (non-photochemical quenching, NPQ), by scavenging free radicals and protecting membranes from lipid peroxidation (Sun et al., 2018). The regulation of NPQ is intrinsically linked to the xanthophyll cycle, such as VAZ cycle and lutein epoxide (LxL) cycle (García-Plazaola et al., 2012). The VAZ cycle is the predominant xanthophyll cycle in most model plants, whereas many neotropical and woody plant species use the LxL cycle (Deng et al., 2017). Here, in the LxL cycle, we found that the content of lutein was reduced and the expression levels of *LUT1* were decreased in *yl* mutant. Mutation of *AtLUT1* can result in *yl* color with limited impact on the growth defect and carotenoids compositions in *Arabidopsis* (Li et al., 2003). The *lut1* mutant of *Arabidopsis* presents remarkable flexibility of plant photosystems with regard to carotenoids compositions. Together with our results, we proposed that the LxL cycle was impaired and results in *yl* color *via* the down expression of *LUT1* in *yl* mutant. In the VAZ cycle, the key regulator genes *ZEP* and *VED* were not obviously effected, whereas the accumulation of zeaxanthin, antheraxanthin, and violaxanthin were remarkably reduced in *yl* mutant. Meanwhile, the expression of ABAs biosynthesis related genes, such as *AOG* and *NCED* were also significantly depressed in *yl* mutant (Figures 3, 7). Together with our former results that the photodamage will occur in mid noon in summer, we implied that the stress tolerance of *yl* mutant may impair during adverse environment, such as in high light and drought stress conditions.

In summary, the carotenoids compositions were altered in *yl* color mutant through regulatory biosynthesis genes. MEP pathway was enhanced to provide more carbon source in plasmids to synthesize more diverse metabolites, such as ubiquinones and tocopherols. Although the xanthophyll cycle was weakened, other metabolism pathways may be evoked for adaptive different environment in *yl* mutant.

### Transcription Factors in Wild-Type and *yl* Plants

A total of 147 DEGs encoding putative transcription factors (TFs) were identified between *yl* and WT (Figure 4C and Supplementary Table S9). They mainly categorized into eight different families: NACs, WRKYs, bHLHs, ERFs, MYBs, C2H2s, GRASs, and bZIPs (Figure 4C). NAC family transcription factor was the most abundant (18 TFs, 12%), where 15 TFs has exhibited upregulated expression in *yl*, followed by the WRKY (16 TFs, 11%), bHLH (13 TFs, 9%), MYB (13 TFs, 9%), and ERF (8 TFs, 5%) (Figure 4C and Supplementary Table S9). Especially, the bHLH, NAC, and MYB transcription factors had reported participation in regulating the synthesis of primary metabolites, such as chlorophyll and carotenoids (Roberts and Susan, 2007; Vranová et al., 2012). Additionally, they may play a critical role on *yl* leaf formation in *R. beggeriana* ‘Aurea.’

## Discussion

Genetic changes usually cause various leaf color types. The direct cause of leaf discoloration is the change in the relative content of plant pigments, such as chlorophylls and carotenoids. In this study, the yellow-green color of the mutant cannot be recovered to normal green and can be inherited stably, indicating that it is genetic controlled.

Chloroplast is the factory of photosynthesis and pigment biosynthesis. In higher plants, the change of a nuclear genes may affect the development and assemble of chloroplasts, and then impact on photosynthesis and abnormal leaf color (Yang et al., 2015). The development of chloroplasts requires the coordination of chloroplast genes and nuclear genes. PPR protein is the key component of the editosome, which was responsible for chloroplast RNA editing site recognition and binding. In *Arabidopsis*, a DYW-type PPR-protein that named *AtECB2* had an interaction with the chlorophyll biosynthetic enzyme porphobilinogen deaminase HEMC and severely affected light-harvesting complex accumulation (Huang et al., 2017). Chloroplast ribosomal protein genes (RP), such as *RPL* and *PRS* play intrinsic roles in the structural stability of the ribosomal complex (Ishii et al., 2006). They are also crucial for the assembly and development of chloroplast (Degenhardt and Bonham-Smith, 2008). In rice, an overexpression of *RPL6* showed an increasing of chlorophylls accumulation and photosynthetic efficiency (Moin et al., 2021). In our study, a number of chloroplastic location PPR proteins were obviously altering in *yl* mutant, implied that chloroplast development defect may be caused by plasmid–nuclear interaction genes (Figure 3 and Supplementary Table S8). Besides, we found 16 DEGs that annotated as heat shock proteins (HSPs) in the “protein processing in endoplasmic reticulum” pathway and most of them had considerably increased in mutated leaves. These HSPs mainly participated in the protein folding, assembly, and maintain the cell homeostasis and played vital roles in the chloroplast development and photosynthesis under heat stress in many species (Wang et al., 2004; Timperio et al., 2008; Waters, 2013). Therefore, we presumed that the overexpression of HSPs in *yl* might be able to maintain the stability of chloroplast structure and enhanced the photochemical efficiency of PSII (*F*_*V*_/*F*_*M*_) to resist heat stress.

Stoma is an important gateway for gas and water exchange between plants and the external environment, which has played a fundamental role in the regulation of plant physiological processes, such as photosynthesis and transpiration. Plants can respond to the stimulation of the external environment by adjusting the opening and closing of stomata. This study showed that the density of stomata in the leaf epidermis of mutated leaves was dramatically reduced comparison to this in WT leaves. Therefore, the reduction of stomata will seriously affect the normal physiological activities in *yl* mutant leaves. For example, slowing response to external adverse stimuli, reducing their stress resistance, and being easy to be burned by sunlight or invaded by diseases and pests.

The leaf color is closely related to the ratio of pigment types and chloroplast status, yellow leaf mutants usually showed the low level of pigment content and photosynthetic efficiency (Gao et al., 2020; Zhang et al., 2020a). Therefore, we paid more attention to photosynthetic system, such as the porphyrin and chlorophyll metabolism, chloroplast development, carotenoid biosynthesis, and photosynthesis-antenna proteins. In *A. thaliana*, the chlorophyll synthesis pathway has been relatively clear, and *SIRB* is the important gene involved in the biosynthesis of siroheme. As a branched pathway of modified tetrapyrroles, the biosynthesis of siroheme is a competitor in synthetic precursors for chlorophyll a and b (Bali et al., 2011). In this study, the transcription levels of *HEMC*, *HEME*, *CHLI*, *DVR*, and *POR* genes that involved in chlorophyll synthesis had all downregulated expression in *yl* mutant (Figures 3, 6A). *NYC1* and *NOL* involved in chlorophylls degradation were also significantly down-expressed (Figures 3, 6C). Both the biosynthesis and degradation of chlorophylls were depressed, and then reduced the accumulation of chlorophylls in *yl* mutant. On the contrast, the expression of *SIRB* genes that involved in siroheme synthesis was up-regulated in *yls* compared with those in green leaves (Figures 3, 6B). The increase expression levels of *SIRB* may cause the reduction of the chlorophyll a/b biosynthesis and accumulation in *yl* mutants, and then resulted in Chlorophyll-deficient mutants formation.

Chlorophyll-deficient mutants are often accompanied by a decrease in light-harvesting proteins (LHC) in the thylakoid membranes of the chloroplast (Highjcin et al., 1969; Polle et al., 2000). LHC proteins are crucial for photosynthesis in plants, which captured light energy and transferred to the reaction centers. In *Arabidopsis*, the lack of *LHCB1* will reduce light absorption, and decrease the efficiency of energy transfer from LHCII to the reaction centers of PSII (Andersson et al., 2003). The depressed of those genes will impaired the function of the complexes, and photosynthesis is inhibited (Tian et al., 2011). Since these complexes are in the thylakoid membrane of chloroplast, they will also disrupt the development of thylakoids (Yang et al., 2015). In PSII, the lack of *PsbP* and *PsbQ* will inhabit the oxygen evolving activity, cell proliferation, and photosynthesis (Haddy et al., 2018; Zienkiewicz et al., 2018). In tobacco, the receptor-side proteins (PsbL, PsbQ, and PsbR), the core proteins of the PSI reaction center (psaA, psaB, psaC, and psaD), electron transport-related proteins (cytb6/f complex, PC, Fd, and FNR), and ATPase subunits are all decreased significantly in Chlorophyll-deficient mutants (Zhang et al., 2020b). In our research, all DEGs encoding PSI, PSII subunits, and ATP synthase showed lower mRNA levels in *yl*. These results are consistent with the decrease in chlorophyll fluorescence parameters and poorly stacked grana in our former study. So we supposed that, the changes in the expression level of photosynthesis genes might account for the reduced photosynthesis capacity in the *yl* mutant.

Carotenoids are the basic components of all photosynthetic organisms and are fat-soluble pigments embedded in the membranes of chloroplasts and colored bodies. In plants, carotenoids can protect chlorophyll from photo-oxygen damage caused by strong light (Bartley and Scolnik, 1995). At the same time, it is an important part of the antenna system and reaction center chlorophyll binding protein, among them, PSI is rich in β-carotene and PSII is rich in xanthin (Niyogi, 1999). All carotenoids are synthesized through isoprenoid or terpenoid pathways. IPP is the precursor of this pathway, PSY, phytoene desaturase (PDS), ZDS, and carotene ε-monooxygenase (LUT1) are the main restriction enzymes, which regulate the synthesis of carotenoids (Giuliano et al., 2008). Impediment in any of those gene’s expression may affect the synthesis of carotenoids. Especially PSY, which is the core enzyme that determines the total amount of carotenoid accumulation in plant. If the gene expression of *PSY* was blocked, will result in the obstruction of carotenoid synthesis and affect the normal accumulation of carotenoids (Burkhardt et al., 2010; Toledo-Ortiz et al., 2010). Except for several carotenoids, such as phytoene, which is colorless, most carotenoids are yellow, red orange, or red (Goodwin, 1992), they are the main color of fruits: mango (*Mangifera indica* L.) (Liang et al., 2020), orange (*Citrus*) (Multari et al., 2020), and tomato (*Lycopersicon esculentum* Mill.) (Akpolat et al., 2020). For many yellow leaf mutant plants, their carotenoid and chlorophyll contents dropped to a very remarkable level. However, the decrease of carotenoid content was less than that of chlorophyll content. The carotenoid/chlorophyll ratio was noticeably decreased, indicating that the carotenoid content increased correspondingly, this might be the basic reason for yellow leaf mutation (Staehelin, 1994).

Transcription factors (TFs) are important regulator that can regulate the spatiotemporal specific expression of target genes by activating or repressing their transcriptional activity, thereby affecting or controlling many molecular functions and biological processes (Mushtaq et al., 2016). We found that among the entries in the molecular function category, the nucleic acid binding transcription factor activity was most significantly enriched, such as NAC, MYB, AP2/ERF, and bHLH. They play an important role in the plants development and in responding to biotic and abiotic stress (Riechmann and Meyerowitz, 1998; Groszmann et al., 2010; An et al., 2012; Lv et al., 2016; Li et al., 2020). *AtMYB2*, *AtMYB15*, *AtMYB41*, *AtMYB44*, *AtMYB61*, *AtMYB102*, and *AtMYBL* are anti-inverse-related MYB transcription factor gene cloned in *Arabidopsis*. *AtMYB2* is a drought induced gene, which encodes R2R3-MYB transcription factor. When plants are subjected to drought stress, it responds to the changes of growth environment by activating the transcription of some ethylene induced genes (Urao et al., 1996; Abe et al., 2003). *AtMYB41* is a gene induced by drought, ABA, and salt stress. It may respond to various stresses by regulating epidermal synthesis and cell proliferation (Lippold et al., 2009; Cominelli et al., 2010; Hoang et al., 2012). *Arabidopsis ERF1* gene can participate in JA, ET, and ABA signal transduction pathways and activate the expression of stress resistant genes. Compared with the control plants, the resistance of overexpressing *ERF1* transgenic *Arabidopsis* to drought, high salt, and high temperature are significantly improved, and the transgenic plants can reduce leaf water loss by reducing stomatal aperture (Cheng et al., 2013). In addition, the transcription factors AP2/ERF, bHLH, MYB, NAC, WRKY, and bZIP in plants are also related to the metabolism of terpenoids and can regulate the transcription level of secondary metabolite synthesis pathway genes (Xu et al., 2019). An overexpression of bHLH transcription factor *PIF5* in *Arabidopsis* showed that the content of tetraterpenoids carotenoids increased (Mannen et al., 2014), and *AabZIP1* can combine with the ABA response element (ABRE) in the promoter region of *ADS* and *CYP71AV1*, the key structural genes of artemisinin biosynthesis pathway, to promote artemisinin biosynthesis (Zhang et al., 2015). The formation and development of stoma is regulated by transcription factors, such as bHLH transcription factors, MYB transcription factors, and Dof transcription factors. When the Dof-type transcription factor gene SCAP1 is knocked out in *A. thaliana*, guard cells appear irregularly distributed, losing the CO_2_-mediated stomatal closure and light-induced stomatal opening function, resulting in the inhibition of stomatal opening and closing (Negi et al., 2013). Therefore, we speculate that the transcription factors screened by transcriptome sequencing may control the production of secondary metabolites by regulating the gene synthesis frequency in phenylalanine metabolic pathway and terpene anabolic pathway, so as to improve the adaptability of mutants to the environment.

## Conclusion

**FIGURE 8:**
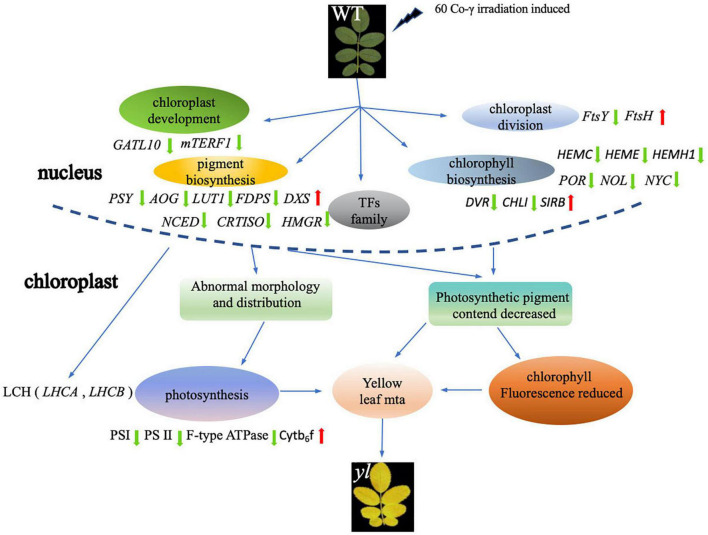
Overview of possible formation pathway of *yls* of the *R. beggeriana*.

In conclusion, the comparison analysis of transcription between WT and *yl* showed that a total of 20,619 unigenes were assembled and 3,372 DEGs were identified. In the *yl* mutation, the expressions of key enzyme genes in the chlorophyll and carotenoid synthesis pathway are attenuated, which directly lead to a decline in pigments. Meanwhile, most of DEGs participated in the chloroplast development and division, photosynthesis is downregulated, which affected chloroplast development and decreased photosynthetic efficient, at last forming different leaf colors. On the other hand, to defend against adverse situations (e.g., high light stress and cold stress), numbers of TFs play crucial roles in the regulation of metabolism, growth, and development, and responses to biotic and abiotic stresses were upregulated (Figure 8). The antioxidant capacity of *yl* mutation has been improved by activating lipid metabolism and promoting the synthesis of antioxidant-related enzymes and more diverse metabolites have been synthesized to adapt the environment by the enhancement of MEP pathway. In addition, *yl* mutant reduced the stomata density to regulate the photosynthesis and evapotranspiration of the leaves to alleviate photodamage. These results provide us more understanding about the regulation mechanisms of plant leaf color formation and the applications of leaf color mutants in the future.

## Data Availability Statement

The datasets presented in this study can be found in online repositories. The names of the repository/repositories and accession number(s) can be found below: NCBI BioProject, PRJNA798584.

## Author Contributions

YG carried out the experiments, performed the data analysis, and drafted the manuscript. YK participated in the data analysis, manuscript revising, and editing. FY, XW, HW, XS, MZ, XZ, and RJ partly participated in the experiments and the data analysis. HG and SY supervised, conceived, and designed the experiments. All authors have seen and approved the final version of the manuscript.

## Conflict of Interest

The authors declare that the research was conducted in the absence of any commercial or financial relationships that could be construed as a potential conflict of interest.

## Publisher’s Note

All claims expressed in this article are solely those of the authors and do not necessarily represent those of their affiliated organizations, or those of the publisher, the editors and the reviewers. Any product that may be evaluated in this article, or claim that may be made by its manufacturer, is not guaranteed or endorsed by the publisher.

## Footnotes

1
https://www.ncbi.nlm.nih.gov/genome/11715?genome_assembly_id=366730

## Supplementary Material

Supplementary Figure S1 Pigment contents in leaves of wild type and yellow-green leaf mutant.

Supplementary Figure S2 The leaf epidermal structure and chloroplast.

Supplementary Figure S3 Parameter details of leaf epidermal structure and chloroplast ultrastructure of wild type and yellow-green leaf mutants.

Supplementary Figure S4 Leaf of wild type and yellow-green leaf mutants.

Supplementary Table S1 The contents of chlorophyll fluorescence of wild type and yellow-green leaf mutant.

Supplementary Table S2 List of primers used for qRT-PCR analysis.

Supplementary Table S3 The quality analysis of all reads from 6 samples.

Supplementary Table S4 All unigenes annotation information from different public databases.

Supplementary Table S5 All DEGs.

Supplementary Table S6 GO class.

Supplementary Table S7 KEGG_enrichment.

Supplementary Table S8 Key DEGs.

Supplementary Table S9 Transcription factors.
