# Supplementary material for: Comparative Transcriptome Profiling Analysis Reveals the Adaptive Molecular Mechanism of Yellow-Green Leaf in Rosa beggeriana ‘Aurea’
Source: Front Plant Sci. 2022 Mar 24;13:845662. doi: 10.3389/fpls.2022.845662 (PMC8987444; doi:10.3389/fpls.2022.845662)
Supplement: Supplementary Figure S1 — Pigment contents in leaves of wild type and yellow-green leaf mutant. [file Presentation_1.zip › supplementary material/Figure S1. Pigment contents in leaves of wild type and yellow-green leaf mutant.docx]

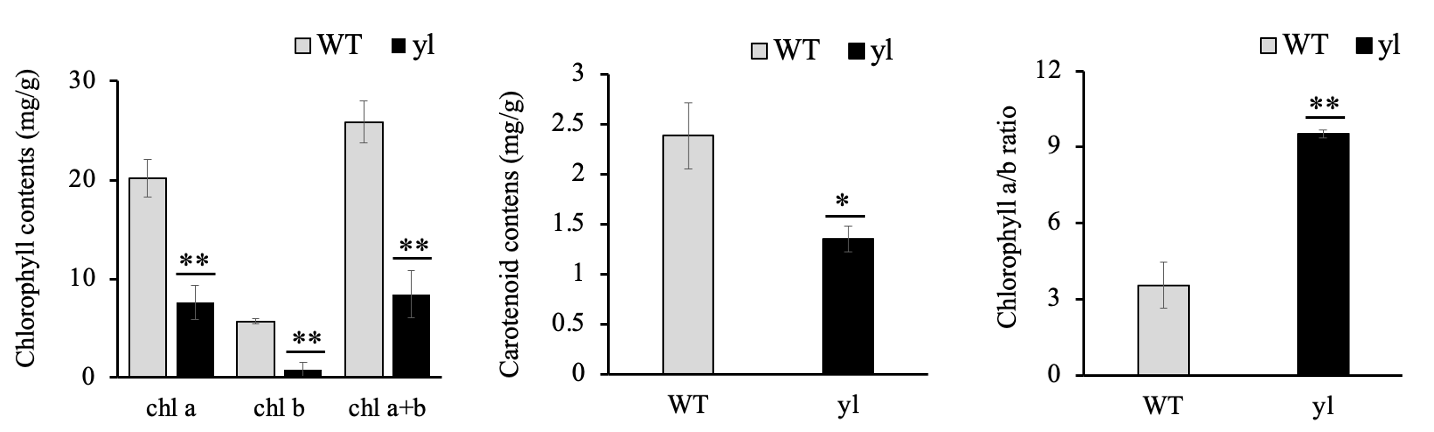


Figure S1. Pigment contents in leaves of wild type and yellow-green leaf mutant. Note: ∗∗ indicates very significant difference of gene expression at 0.01 level, ∗ indicates significant difference of gene expression at 0.05 level. WT present wild-type *R. beggeriana*, *yl* present mutant *R. beggeriana* ‘Aurea’.
