# Supplementary material for: Comparative Transcriptome Profiling Analysis Reveals the Adaptive Molecular Mechanism of Yellow-Green Leaf in Rosa beggeriana ‘Aurea’
Source: Front Plant Sci. 2022 Mar 24;13:845662. doi: 10.3389/fpls.2022.845662 (PMC8987444; doi:10.3389/fpls.2022.845662)
Supplement: Supplementary Figure S1 — Pigment contents in leaves of wild type and yellow-green leaf mutant. [file Presentation_1.zip › supplementary material/Figure S2. The leaf epidermal structure and chloroplast ultrastructure of wild type and yellow-green leaf mutant.docx]

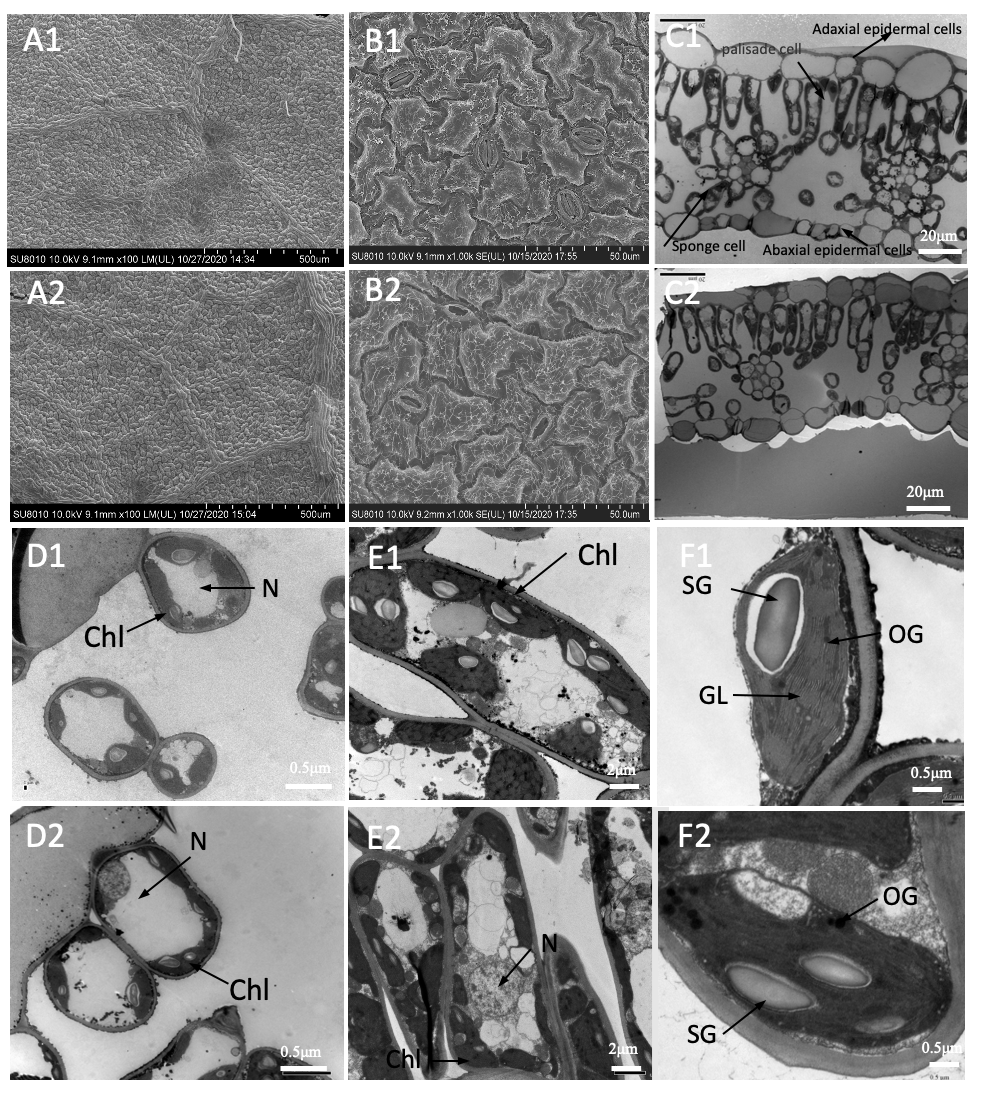


Figure S2. The leaf epidermal structure and chloroplast ultrastructure of wild type and yellow-green leaf mutant. A1-E1 present wild type, A2-E2 present yellow-green leaf mutant. A1, A2: the upper epidermis; B1, B2: The lower epidermis; C1, C2: Leaf transmission electron micrographs of cross sections; D1, D2: the spongy parenchyma of the mesophyll cells; E1, E2: the palisade parenchyma of the mesophyll cells; F1, F2: chloroplast. N: Nucleus; SG: starch grain; GL: grana lamella; OG: osmiophilic granule.
