# Supplementary material for: Comparative Transcriptome Profiling Analysis Reveals the Adaptive Molecular Mechanism of Yellow-Green Leaf in Rosa beggeriana ‘Aurea’
Source: Front Plant Sci. 2022 Mar 24;13:845662. doi: 10.3389/fpls.2022.845662 (PMC8987444; doi:10.3389/fpls.2022.845662)
Supplement: Supplementary Figure S1 — Pigment contents in leaves of wild type and yellow-green leaf mutant. [file Presentation_1.zip › supplementary material/Figure S3. Parameter details of leaf epidermal structure and chloroplast ultrastructure of wild type and yellow-green leaf mutants..docx]

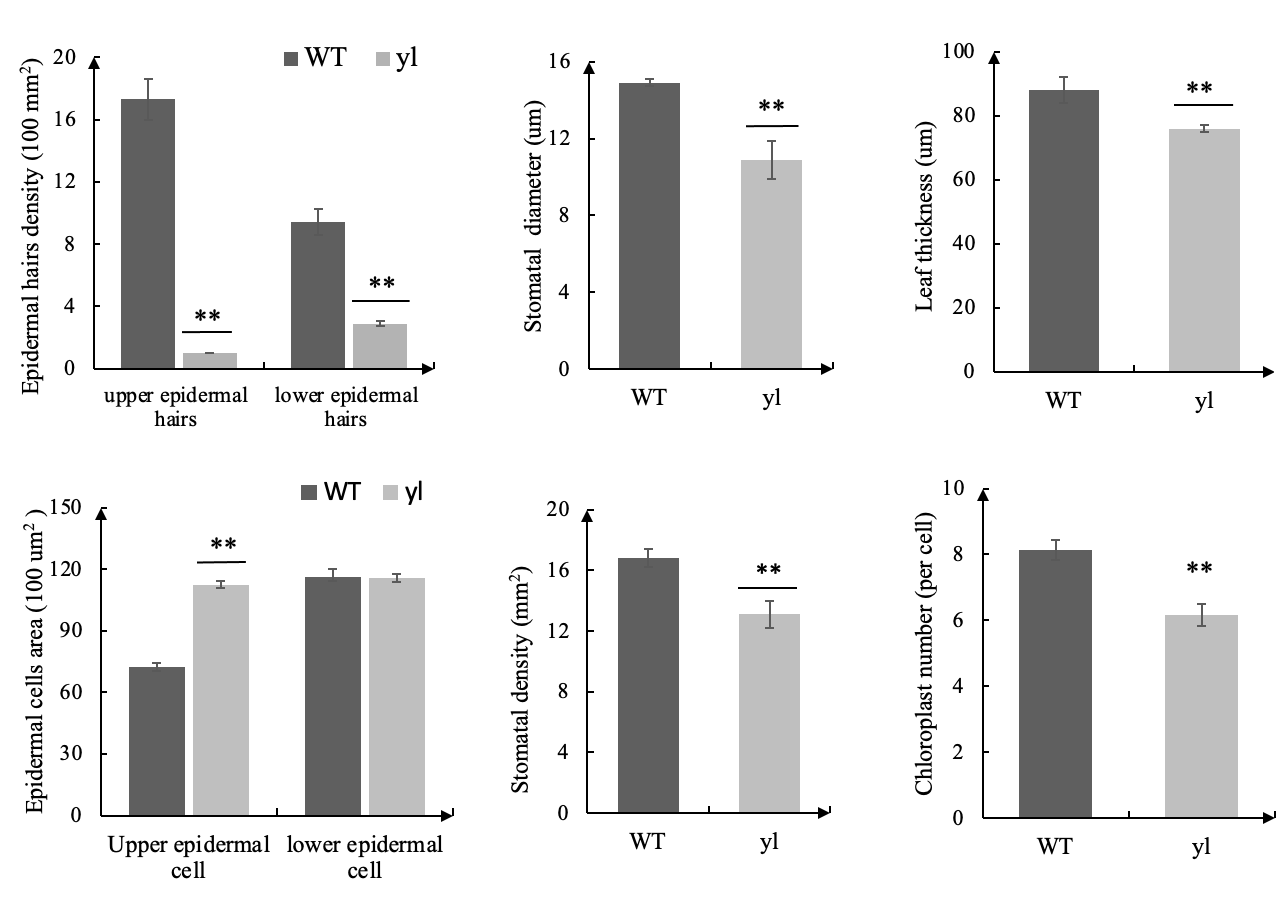
 Figure S4. Parameter details of leaf epidermal structure and chloroplast ultrastructure in wild-type and yellow-green leaf mutants.
