# Supplementary material for: Comparative Transcriptome Profiling Analysis Reveals the Adaptive Molecular Mechanism of Yellow-Green Leaf in Rosa beggeriana ‘Aurea’
Source: Front Plant Sci. 2022 Mar 24;13:845662. doi: 10.3389/fpls.2022.845662 (PMC8987444; doi:10.3389/fpls.2022.845662)
Supplement: Supplementary Figure S1 — Pigment contents in leaves of wild type and yellow-green leaf mutant. [file Presentation_1.zip › supplementary material/Figure S4. Leaf of wild type and yellow-green leaf mutants.docx]

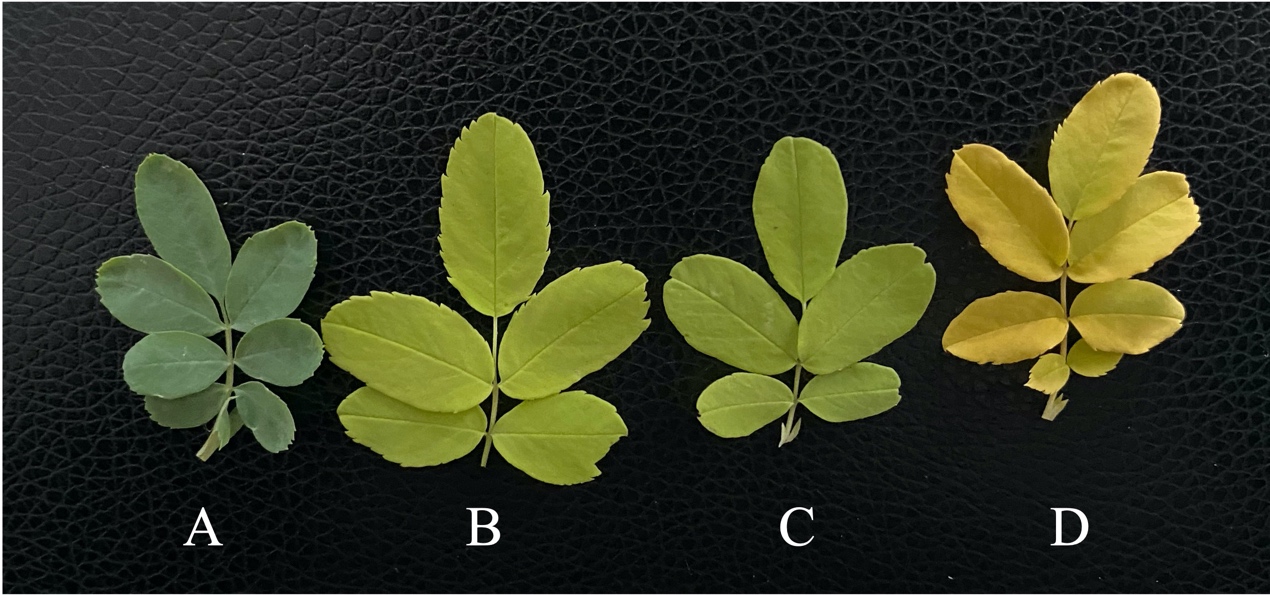


Figure S4. Leaf of wild type and yellow-green leaf mutants. A: Normal green leaf color from wild type plants. B, C: Yellow-green color leaves from *yl* mutant under shading conditions (Lower location in plant). D: Yellow-green color leaves from *yl* mutant (Upper location in plant). The yellow-green color leaves from the same mutant plant would exhibit more green color under shading conditions (Fig S4B,C).
