# Supplementary material for: Comparative Transcriptome Profiling Analysis Reveals the Adaptive Molecular Mechanism of Yellow-Green Leaf in Rosa beggeriana ‘Aurea’
Source: Front Plant Sci. 2022 Mar 24;13:845662. doi: 10.3389/fpls.2022.845662 (PMC8987444; doi:10.3389/fpls.2022.845662)
Supplement: Supplementary Figure S1 — Pigment contents in leaves of wild type and yellow-green leaf mutant. [file Presentation_1.zip › supplementary material/Supplementary material list.docx]

**Supplementary**

**Figure S1.** Pigment contents in leaves of wild type and yellow-green leaf mutant.

**Figure S2.** The leaf epidermal structure and chloroplast.

**Figure S3.** Parameter details of leaf epidermal structure and chloroplast ultrastructure of wild type and yellow-green leaf mutants.

**Figure S4.** Leaf of wild type and yellow-green leaf mutants.

**Table S1.** The contents of chlorophyll fluorescence of wild type and yellow-green leaf mutant.

**Table S2.** List of primers used for qRT-PCR analysis.

**Table S3.** The quality analysis of all reads from 6 samples.

**Table S4.** All unigenes annotation information from different public databases.

**Table S5.** All DEGs.

**Table S6.** GO class.

**Table S7.** KEGG_enrichment.

**Table S8.** Key DEGs.

**Table S9.** Transcription factors.
