# Supplementary material for: Comparative Transcriptome Profiling Analysis Reveals the Adaptive Molecular Mechanism of Yellow-Green Leaf in Rosa beggeriana ‘Aurea’
Source: Front Plant Sci. 2022 Mar 24;13:845662. doi: 10.3389/fpls.2022.845662 (PMC8987444; doi:10.3389/fpls.2022.845662)
Supplement: Supplementary Figure S1 — Pigment contents in leaves of wild type and yellow-green leaf mutant. [file Presentation_1.zip › supplementary material/Table S1. The contents of chlorophyll fluorescence of wild type and yellow-green leaf mutant.docx]

|  | **Fv/Fm** | **Y(II)** | **ETR(II)** | **Y(NO)** | **Y(NPQ)** | **NPQ** | **qN** | **qP** | **qL** |
| --- | --- | --- | --- | --- | --- | --- | --- | --- | --- |
| **WT** | 0.71±0.02b | 0.44±0.03a | 41.98±2.84a | 0.23±0.01a | 0.32±0.02b | 1.38±0.12b | 0.70±0.02b | 0.88±0.02a | 0.78±0.03a |
| ***yl*** | 0.65±0.01a | 0.49±0.02a | 45.90±1.76a | 0.35±0.01b | 0.16±0.02a | 0.46±0.08a | 0.40±0.05a | 0.87±0.01a | 0.76±0.01a |

Table S2. The contents of chlorophyll fluorescence of wild type and yellow-green leaf mutant

Note: Values are means ± SE with three biological replicates. In each column, means followed by different letters indicate significant differences at *P*<0.05.
