# Supplementary material for: Comparative Transcriptome Profiling Analysis Reveals the Adaptive Molecular Mechanism of Yellow-Green Leaf in Rosa beggeriana ‘Aurea’
Source: Front Plant Sci. 2022 Mar 24;13:845662. doi: 10.3389/fpls.2022.845662 (PMC8987444; doi:10.3389/fpls.2022.845662)
Supplement: Supplementary Figure S1 — Pigment contents in leaves of wild type and yellow-green leaf mutant. [file Presentation_1.zip › supplementary material/Table S2. List of primers used for qRT-PCR analysis..docx]

**Table S1. List of primers used for qRT-PCR analysis.**

| Trinity ID | Primer Name | Sequence |
| --- | --- | --- |
| RcHm_v2.0_Chr1g0352821.gene | *RbFTSY*-F | GCTGGAGATATGGGCTGA |
|  | *RbFTSY*-R | GTTCTTTCCCTCTCTTCACAGC |
| RcHm_v2.0_Chr4g0439481.gene | *RbPSBQ*-F | TTGGTGCCGGAAGTTAACCG |
|  | *RbPSBQ*-R | CATACACCTTTGGCGTCCGA |
| RcHm_v2.0_Chr2g0151701.gene | *RbLHCB1*-F | ACTTGGGCCCATTCTCCTGT |
|  | *RbLHCB1*-R | ATGGCCCACCTGGAATGGAT |
| RcHm_v2.0_Chr5g0046981.gene | *RbPSAO*-F-R | GAAGCTCGCTCTCGTCCAAG |
|  | *RbPSAO*-R | GGTGAACCTTCCTCCAGAAGC |
| RcHm_v2.0_Chr4g0421151.gene | *RbDVR*-F | AGTCGTTAAGTTCAGCAAAGACAG |
|  | *RbDVR*-R | AATGTACCCAGTTGCACCCA |
| RcHm_v2.0_Chr2g0096771.gene | *RbHMGCR*-F | TGTCACCATGCCGTCCATTG |
|  | *RbHMGCR*-R | TCCCGGTGCCTCTCTGTTAG |
| RcHm_v2.0_Chr2g0090301.gene | *RbNOL*-F | GCTGTGGCTAAAGTGGAGGA |
|  | *RbNOL*-R | CTGACAACTGAAACCCAATTGCT |
| RcHm_v2.0_Chr1g0371761.gene | *RbHEME*-F | AGGGCTTGAAGACTTTGCATCC |
|  | *RbHEME*-R | ACGTAGCGATTGTCCAAGGC |
| RcHm_v2.0_Chr5g0034621.gene | *RbPOR*-F | CGCGGTTGATGGTTGAGGAT |
|  | *RbPOR*-R | CAAGGTTGGCCTTTGGAGGT |
| RcHm_v2.0_Chr6g0256571.gene | *RbAOG*-F | GGCCTCTGACCTCATCGACT |
|  | *RbAOG*-R | ACCACAAACGGCTCCGAATC |
| RcHm_v2.0_Chr2g0168361.gene | *RbFtsH-F* | GAGAAGTTTGCTGCGGTTGG |
|  | *RbFtsH-R* | CTCCTGCAATGGCCTTACCC |
| RcHm_v2.0_Chr2g0169951.gene | *RbGATL10-F* | AGTTGTCCGCTCCACGTTTC |
|  | *RbGATL10-R* | TCGACGCAGGAGTCTAGCAA |
| RcHm_v2.0_Chr4g0446801.gene | *RbHEMC-F* | ATGCCAGCAAAGATGCCGAT |
|  | *RbHEMC-R* | AACAACCAAAGAAGCCAGGACC |
| RcHm_v2.0_Chr5g0010061.gene | *RbCHLI-F* | TTGCTTGACCGTTTTGGTATGC |
|  | *RbCHLI-R* | TATGTCTCCTCTCAATCCGTCG |
| RcHm_v2.0_Chr2g0095461.gene | *RbLHCA2-F* | GCAGCCTTCCCGGTGATTT |
|  | *RbLHCA2-R* | AGCCTAGTCTTTCAAGCCACTCT |
| RcHm_v2.0_Chr1g0370011.gene | *RbLHCB4-F* | GGGCAATGCTTGGCTCTCTT |
|  | *RbLHCB4-R* | AAGGGAAGTGGAAGACCAAGG |
| RcHm_v2.0_Chr2g0140671.gene | *RbLHCB1-F* | CCACTTGGCTGACCCTGTTA |
|  | *RbLHCB1-R* | GAGAGAGCCATTGTTGAGGAAG |
| RcHm_v2.0_Chr2g0126111.gene | *RbRbcS-F* | CCAAGCAATGGCGGAAGAGT |
|  | *RbRbcS-R* | CAGGGAACCCATCCTTTGCG |
| RcHm_v2.0_Chr3g0464191.gene | *RbFBP-F* | AGAAACTCGACGTCGTCTCCA |
|  | *RbFBP-R* | GCCACTGGCACATCCTCTTC |
| RcHm_v2.0_Chr7g0209601.gene | *RbGAPDH-F* | GATGTTGAGCTCGTCGCTGT |
|  | *RbGAPDH-R* | GGGTCCTTTCGTCCTTGACC |
| RcHm_v2.0_Chr4g0403671.gene | *RbDXS-F* | TTCCGCATCTCCTCCTCACC |
|  | *RbDXS-R* | GGAGTTGAGGGCCTTTCTCCT |
| RcHm_v2.0_Chr7g0179091.gene | *RbGST-F* | CAGGACCCTTGTGACAGAGC |
|  | *RbGST-R* | GCAGCCTCTATTGCCTTCTCC |
| RcHm_v2.0_Chr4g0396391.gene | *RbGST23-F* | TGGCCTGAGAATCCACTGCT |
|  | *RbGST23-R* | CCAATGTCCTCAGCGTTTCCA |
| RcHm_v2.0_Chr1g0330061.gene | *RbCRSP-F* | AGAACTGCAACCAATGTCGGA |
|  | *RbCRSP-R* | ACCTTCCTCACACCTTCAGCA |
| RcHm_v2.0_Chr1g0369161.gene | *RbDOF3.1-F* | GGCGGTGGAAGCAGAAAGAA |
|  | *RbDOF3.1-R* | CAGCTGCTGAGCTTGACAGG |
| RcHm_v2.0_Chr6g0276721.gene | *RbDOF-F* | AAAGGCGGGTCCCTGAGAAA |
|  | *RbDOF-R* | GATCCGTTGGCTCCACCTTC |
| RcHm_v2.0_Chr6g0289711.gene | *RbCRTISO-F* | CATCTGTCTCAAAATGTCCAAG |
|  | *RbCRTISO-R* | CTGAAGAGCTTGAACGACTATT |
| RcHm_v2.0_Chr5g0014331.gene | *RbNCED-F* | CATCGCCTTGAACAGAAATAGA |
|  | *RbNCED-R* | GCGTTGTAGTTACCGTTTTAGG |
| RcHm_v2.0_Chr3g0471231.gene | *RbLUT1-F* | TTACACTGCATTGAAAGAAGCG |
|  | *RbLUT1-R* | CCTCATCAATTCTTTCACCCTC |
| RcHm_v2.0_Chr3g0488531.gene | *RbPSY-F* | ATCTTTTCCAAGGTCGTCCATT |
|  | *RbPSY-R* | CCATTCTCATTCCCTCTATCAT |
