# Supplementary material for: Comparative Transcriptome Profiling Analysis Reveals the Adaptive Molecular Mechanism of Yellow-Green Leaf in Rosa beggeriana ‘Aurea’
Source: Front Plant Sci. 2022 Mar 24;13:845662. doi: 10.3389/fpls.2022.845662 (PMC8987444; doi:10.3389/fpls.2022.845662)
Supplement: Supplementary Figure S1 — Pigment contents in leaves of wild type and yellow-green leaf mutant. [file Presentation_1.zip › supplementary material/Table S3. The quality analysis of all reads from 6 samples.docx]

Table S3 The quality analysis of all reads from 6 samples

| **Samples** | **Clean reads** | **GC Content** | **Q30** | **Mapped Reads** | **Ratio** |
| --- | --- | --- | --- | --- | --- |
| WT-1 | 25,482,425 | 47.79% | 92.57% | 31,013,373 | 60.85% |
| WT-2 | 25,224,982 | 48.02% | 90.11% | 29,816,394 | 59.10% |
| WT-3 | 26,566,098 | 48.08% | 90.01% | 31,708,958 | 59.68% |
| yl-1 | 25,704,990 | 47.62% | 92.71% | 30,991,722 | 60.28% |
| yl-2 | 25,663,216 | 47.72% | 92.79% | 30,639,618 | 59.70% |
| yl-3 | 26,900,353 | 47.29% | 93.00% | 33,356,741 | 62.00% |
| Total | 155,542,064 | 47.75% | 91.87% | 187,526,806 | 60.27% |
